# Robust Single-Trial EEG-Based Authentication Achieved with a 2-Stage Classifier

**DOI:** 10.3390/bios10090124

**Published:** 2020-09-13

**Authors:** Uladzislau Barayeu, Nastassya Horlava, Arno Libert, Marc Van Hulle

**Affiliations:** 1 Department of Biophysics, Belarusian State University, 220030 Minsk, Belarus; baraewlad@gmail.com; 2 Department of Mathematical Modelling and Data Analysis, Belarusian State University, 220030 Minsk, Belarus; nastassya.horlava@mailbox.tu-dresden.de; 3 Laboratory for Neuro- and Psychophysiology, Department of Neuroscience, KU Leuven, O&N2, Herestraat 49, 3000 Leuven, Belgium

**Keywords:** person authentication, EEG, neural network, SVM

## Abstract

The risk of personal data exposure through unauthorized access has never been as imminent as today. To counter this, biometric authentication has been proposed: the use of distinctive physiological and behavioral characteristics as a form of identification and access control. One of the recent developments is electroencephalography (EEG)-based authentication. It builds on the subject-specific nature of brain responses which are difficult to recreate artificially. We propose an authentication system based on EEG signals recorded in response to a simple motor paradigm. Authentication is achieved with a novel two-stage decoder. In the first stage, EEG signal features are extracted using an inception- and a VGG-like deep learning neural network (NN) both of which we compare with principal component analysis (PCA). In the second stage, a support vector machine (SVM) is used for binary classification to authenticate the subject based on the extracted features. All decoders are trained on EEG motor-movement data recorded from 105 subjects. We achieved with the VGG-like NN-SVM decoder a false-acceptance rate (FAR) of 2.55% with an overall accuracy of 88.29%, a FAR of 3.33% with an accuracy of 87.47%, and a FAR of 2.89% with an accuracy of 90.68% for 8, 16, and 64 channels, respectively. With the Inception-like NN-SVM decoder we achieved a false-acceptance rate (FAR) of 4.08% with an overall accuracy of 87.29%, a FAR of 3.53% with an accuracy of 85.31%, and a FAR of 1.27% with an accuracy of 93.40% for 8, 16, and 64 channels, respectively. The PCA-SVM decoder achieved accuracies of 92.09%, 92.36%, and 95.64% with FARs of 2.19%, 2.17%, and 1.26% for 8, 16, and 64 channels, respectively.

## 1. Introduction

Along with the evolution in digital information acquisition and storage is the need for increasingly more advanced authorization systems. Typically, such systems require individuals to produce a highly specific phrase, word, or feature to obtain access. Other bodily parameters and signals have successfully been used for authentication for example fingerprints, iris scans, and writing patterns. This branch of security systems is called biometric authorization [1]. A secure authorization system requires features to be user-related and difficult to simulate [1,2]. In recent works, electroencephalography (EEG) has been suggested as a biometric credential [3,4] due to its subject-specific and unbidden nature. Researchers have used EEG data in both identification and authentication settings. Three measurements can be used to assess the quality of an identification and authentication system namely the accuracy, the False Rejection Rate (FRR) and the False Acceptance Rate (FAR), where FAR is the rate of successful authentication by imposters (unauthorized users) and FRR the rate of denial of registered users.

Identification, where the subject should be correctly identified from a dataset of subjects, is a multiclass classification problem. The multiclass nature of identification makes implementation challenging in a real-world setting. In [5] researchers achieved an impressive 100% accuracy for EEG-based identification of 108 subjects. They used the ‘eyes open’ and ‘eyes closed’ resting states of the subjects combined with functional connectivity patterns. However, they report a lengthy evaluation time to achieve identification. Ruiz-Blondet et al. [6] developed an event-related potential (ERP)-based identification system termed CEREBRE that encoded several individually unique responses from multiple brain systems. They achieved 100% accuracy when using multiple responses and 86% accuracy when using a single response [7]. The complexity of current identification systems hampers their usability as they are computationally too heavy or time consuming [8], a picture that might change with the development of more powerful computational resources and better EEG paradigms and signal features.

Authentication is the verification of the claimed identity of the user [1,2]. In other words, the user claims an identity and the system verifies it. Thus, a binary classification problem. Several studies investigated different approaches to EEG-based authentication. Hu et al. [8] used imagined movement recordings of three subjects. Features were extracted using an Autoregressive-moving-average model (ARMA) and a 5-layer neural network was used for classification. FRR varied from 15% to 25% whilst FAR results were missing. Yeom et al. [4] collected data from 10 users in response to images of their own face (self-face) and that of others (non-self-face). They selected temporal and dynamic features from 18 channels and used a support vector machine (SVM) for classification. They achieved an average FRR of 13.9% and FAR of 13.9%. Wu et al. [9] collected EEG and electro-oculography (EOG) data from 40 subjects (15 users, 25 imposters). A rapid serial visual presentation of faces (face-RSVP) was used for stimulation. They compiled a feature matrix of the average ERPs and used a convolutional neural network for classification. The reported FRR and FAR were 8.49% and 6.23%, respectively. Ashby et al. [10] used an Emotiv Epoch headset for EEG recording, gathering scores for 1358 features for each trial. An SVM was trained using 20% user trials and 80% non-user (imposter) trials. They obtained an accuracy of 100% requiring up to 40 trials for one authentication attempt. Unfortunately, the authors did not report any FAR or FRR results. Mu et al. [11] also used EEG recordings from 16 users with self-face and non-self-face images as stimuli. For feature selection, they used a Fisher-based distance metric method and trained an SVM for classification. An accuracy of 90.7% was achieved. FAR and FRR calculations were not reported. 

In this work, we propose a 2-stage authenticator which we developed using a dataset of EEG responses to a performed and imagined motor task recorded from 105 subjects who performed three experiments with at least seven trials each resulting in 21 trials per subject [12,13]. We used empirical mode decomposition [14] to isolate frequency bands in a data-driven way, computed signal complexity and power per frequency band, and selected various subgroups of task-relevant electrode channels (8, 16, or 64), yielding an 18(features) ×8/16/64(channels) matrix for different subgroups, respectively. The feature matrix was then reduced either by using principal component analysis (PCA), an inception- [15] or a VGG deep convolutional neural network (NN) [16] suited for dimensionality reduction [17]. The reduced feature matrix (18 × 2) was then used to train an SVM for binary classification [18,19]. We aimed to develop a system with an easy to perform, universally performable task whilst being difficult to fool by imposters as required in a real-world setting.

## 2. Materials and Methods

### 2.1. Recording

We used the “EEG Motor Movement/Imagery Dataset” [12] of the publicly available PhysioNet databank [13]. Subjects engaged in cued performed- and imagined movement tasks whilst a 64-channel EEG was recorded using the BCI2000 software system [20]. In total, 109 subjects were recorded of which 4 subjects did not perform 21 trials due to an annotation error, hence, their recordings were not further considered (thus, recordings from 105 subjects are further considered). Each subject had 3 sessions consisting of around 8 trials. A trial consisted of the subject clenching or tightening the displayed target hand (left or right hand) into a fist followed by the relaxation or unclenching of the hand. Some subjects only performed 7 trials; hence 21 trials were used for each subject. Prior to the trials, subjects performed a one-minute baseline run with eyes open and a one- minute baseline run with eyes closed. Note that an authentication system is developed for each subject individually.

### 2.2. Preprocessing

The training of deep learning NN applications call for a substantial number of data entries [15]. To increase the amount of trials in our case, we applied sliding windows with a length of 2 s and 75% overlap. In total, this resulted in around 105 trials per subject for each task (thus, going from 21 to 105 trials). The EEG signal was subsequently filtered using zero phase delay with cutoff frequencies at 1 and 50 Hz. All preprocessing was performed in MATLAB. 

### 2.3. Feature Extraction Methods

To assess signal complexity across frequency bands, we used Empirical Mode Decomposition [14]. A given signal can be represented as a sum of modulated components known as Intrinsic Mode Functions (IMFs):

(1) Signal=IMF1+IMF2+⋯+IMFn+Residue.

**Figure 1:**
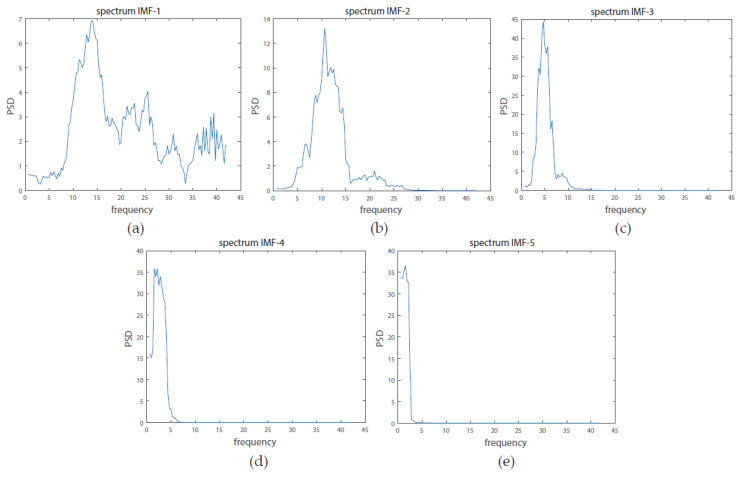
Example of Power Spectral Density (PSD) of the first 5 Intrinsic Mode Functions (IMFs) of one trial from one of the users. (**a**–**e**) respectively represent the first 5 calculated IMFs.

Each IMF is defined in a data-driven way and corresponds to a partially overlapping frequency band. The EMD was calculated with 40 dB resolution and 60 dB residual energy as stop criteria [21]. From each IMF, the Power Spectral Density (PSD) is calculated using the multi-taper method of the Chronux toolbox [22]. After inspecting the results (Figure 1), we concluded that most information is present in the first 4 IMFs. Henceforth, all subsequent processing was performed with these IMFs. 

For each channel and IMF we calculated the univariate Shannon entropy [23], log entropy [24], sample entropy [25], and approximate entropy [25,26]. Shannon entropy *S* is given by the formula:

(2) S=−∑p(x)log(p(x)),

log energy *L* by the formula:

(3) L=∑log(p(x)),

where *p*(*x*) is the probability of character *x* appearing in the stream of characters of the message. Approximate Entropy (ApEn) is given by the formula:

(4) $$ApEn\left(r,m,N\right)=-\log\left(\frac{A^{m\;+\;1}\left(r\right)}{B^{m}\left(r\right)}\right),$$

and Sample Entropy (SampEn) by the formula:

(5) $$SampEn\left(r,m,N\right)=-log\frac{A^{m\;+\;1}\left(r\right)}{B^{m}\left(r\right)},$$

with *B^m^* the probability that 2 sequences with length m are similar within tolerance level r, *A^m^*^+1^ the probability that the same 2 sequences are again similar within tolerance level r when their length is extended to *m* + 1. Note that the calculation of ApEn includes self-matching, whilst SampEn does not. Here, *m* = 2 and r was set as 15% of the standard deviation of the time-series.

Two additional features for each of the channels were the average powers of the mu (7.5–12.5 Hz) and beta (16–31 Hz) rhythms expressed by the Power Spectral Density (PSD). PSD was calculated with the multitaper method on the Chronux toolbox [22]. In total 18 features were obtained for each channel.

### 2.4. Channel Selection

Channel selection in accordance with task-related regions on the scalp can positively influence the performance of the EEG-based authentication system as well as reduce the EEG setup- and system training times which are deemed important in a real-life setting. Hence, we performed a comparison of 3 different systems utilizing data from the following channels in accordance with the international 10–20 system:

- 8 channels: {Fz, FCz, C3, C4, F1, F2, AF3, AF4};
- 16 channels: {Fz, FCz, C3, C4, F1, F2, AF3, AF4, F4, Fp1, Fp2, C1, C2, FC1, FC2, F3};
- all 64 channels

Figures of the recording locations can be found in the supplementary materials (supplementary Figure S6). The 8 and 16 channels were chosen in accordance with the scalp topography of motor function.

### 2.5. Feature Dimensionality Reduction

**Figure 2:**
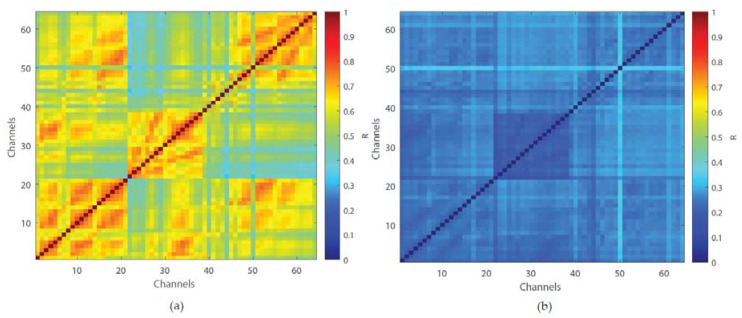
Mean cross-correlation of all features (**a**) and standard deviation of the mean (**b**).

Feature extraction resulted in an 18 × (8, 16 or 64) matrix, depending on which channels were selected. To reduce the number of features, we conducted multichannel analyses using the cross-correlation method [27] and noted 2 clusters of channels. For each feature, we calculated the mean and standard deviation of the cross-correlation between all pairs of channels. The cross- correlation results of the 18 × 64 matrix are shown in Figure 2. Cross-correlation results of 18 × (8 and 16) can be found in supplementary Figure S5. Concatenation of the channels transforms the 18 × 2 matrix into a 36 × 1 matrix which is the input to the SVM classifier (see further).

### 2.6. Neural Network Architectures

For dimensionality reduction and feature selection we used a neural network which performed channel convolution, developed with the Keras framework in Python [28]. We used a set of convolutions to unveil the dependencies within each feature, whilst not affecting the dependencies between them. To this end, convolutional layers with 1×n-type kernels and 1×m strides were used across the network. The convolutional layer used linear activation, followed by batch normalization to avoid overfitting, after which the Rectified Linear Unit (ReLU) function was applied. Two types of architecture were developed, an inception-like variant and a VGG-like variant.

**Figure 3:**
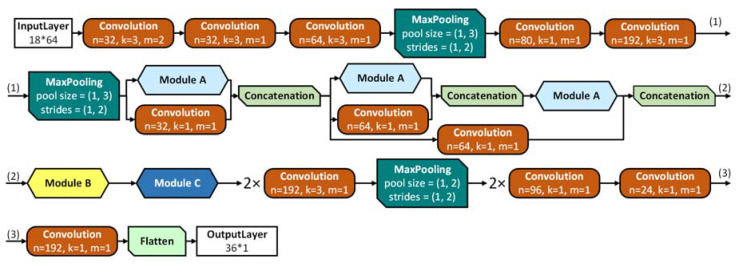
Representation of the full inception-like neural network (NN) for 64 channels. The input matrix of 18 × 64 was converted by the NN into an 18 × 2 matrix and concatenated into a 36 × 1 by a flattening layer. The first layers are traditional sets of convolutions, followed by pooling layers for grid reduction. Next, a sequence of 3 modules of type A was applied, followed by modules of type B and C. For the first two A modules, additional convolutions were concatenated to keep the balance between network width and depth. An additional convolution was added to concatenate the output of the first and third module A to demote the vanishing gradient problem. For 8 and 16 channels the structure was similar with the strides decreased to 8 and 16 convolutions, respectively.

The first architecture was based on the idea of inception-like modules [15] which combine convolutions with different kernels in the same layer and thus acts as a small network within the overarching one. These modules allowed for a deep network whilst avoiding the vanishing gradient problem [15,29], by keeping the balance between the width and depth of the network. A detailed structure of the inception-like architecture is represented in Figure 3, the design of the modules can be found in supplementary Figures S1 and S2.

**Figure 4:**
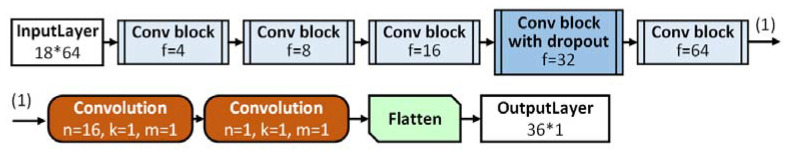
Structure of the VGG-like NN. Note the usage of kernels with size 1 × 3 and 26 of several consecutive convolutions with the same specifics before pooling.

In [30], the authors concluded that the most successful neural network EEG applications were achieved by means of shallow networks. Hence, we designed a second architecture based on the VGG neural network [16,31]. Its structure is presented in Figure 4 and in supplementary Figure S3.

Both architectures were adapted for feature selection in 64, 16, and 8 channels. To adapt the network to the first stage of our authentication system, training was considered as a transfer problem. Hereto, we expanded both architectures with a tail consisting of a set of dense layers combined with a dropout layer (see the supplementary material). The expanded networks were subsequently trained using the Adam optimization method [32] for 350 epochs with a learning rate of 10^−3^ for convergence, followed by another 350 epochs with learning rate 10^−4^. Afterwards, the tail was removed whilst the weights were used for the first stage of the decoder, i.e., feature reduction, transforming the input matrix into a 2 × 18 matrix (subsequently flattened into vector 1 × 36).

### 2.7. Principal Component Analysis

As an alternative to the convolution within channels of the neural networks, we considered principal component analysis (PCA) [33] as it transforms a number of possibly correlated variables into a smaller number of uncorrelated ones that capture the largest part of the data variance (eigenvalues). We used PCA to reduce the initial 18 features into 2 for each of the channels used. 

### 2.8. Classification and Grading

For the second stage of the decoder we used an SVM classifier operating on the feature matrix resulting from NN or PCA. We compared the performance between an NN-SVM decoder with input features taken from the SVM from the pre-trained NN algorithms and a PCA-SVM decoder with features taken from PCA (i.e., eigenvectors). The first model combines the pre-trained neural network with SVM (NN-SVM); the second model combines PCA with SVM (PCA-SVM). For training the SVM, both models used the 105 trials of the user and 105 trials from other users (imposters) by randomly selecting a subset of trials from other users. The SVM was implemented with the matlab function fitcsvm using a radial basis function (rbf) kernel, standardization, and automatic kernel scaling.

**Figure 5:**
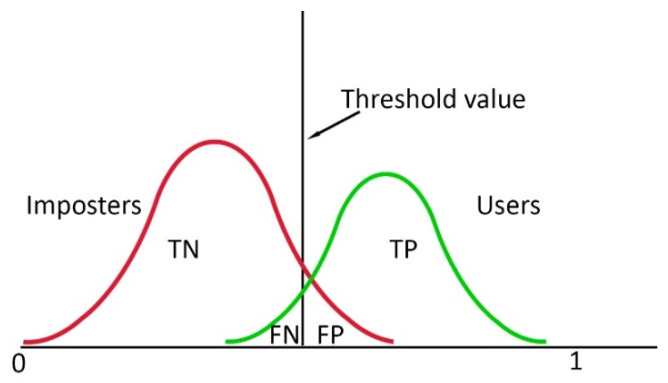
Visual representation of True Positive (TP) being the positive examples classified as positive, True Negative (TN) being the negative examples classified as negative, False Negative (FN) being the negative trials classified as positive, and False Positive (FP) being the positive trials classified as negative given the distributions of authorized individuals (green) and imposters (red).

We determined the accuracy of both models using 5-fold cross validation: all data was sequentially divided into 5 groups (20% for testing and another 80% for training). Note that the validation datasets were part of the training data resulting from the k-fold cross-validation but not part of the test data. In each fold, we normalized our training data for each feature and the test data according to the maximum and minimum of the training data. After that, we calculated the mean accuracy for all test sets across subjects. To represent the authentication system performance, we used the False Acceptance Rate (FAR) and False Rejection Rate (FRR). The FRR is defined as follows:

(6) $$FRR=\sum_{i,j}\frac{FN_{ij}}{TP_{ij}+FN_{ij}},$$

where *i* is the user and *j* is the fold and TP stands for True Positive, the number of correctly granted authentication attempts of the users, and FN for False Negative, the number of incorrectly denied authentication attempts of the users. A visual representation is presented in Figure 3. FAR is defined as follows:

(7) $$FAR=\sum_{i,j}\frac{FP_{ij}}{TN_{ij}+FP_{ij}}$$

where *i* is the user and *j* is the fold and TN stands for True Negative, the number of correctly denied authentication attempts of the imposters, and FP for False Positive, the number of incorrectly granted authentication attempts by the imposters (Figure 5). 

As input for the SVM we used a group of preselected features. The preselection was done as follows: first, for each feature, we calculated the SVM classification accuracy using 5-fold cross validation within the training set. Second, we adopted a forward selection procedure. We selected the top-5 features with the highest individual accuracy. After that, accuracies were calculated for all possible pairs. The top-5 pairs with highest accuracies were eventually retained. We continued this process until the optimal group of 10 features was obtained; we reported the classification results for this preselected group of features. Note that all systems had the same number of features in the prediction, the increased number of channels provided additional information but did not increase the number of features in the SVM.

We argue that in an authentication system it is most important not to admit an imposter, a 2-task classifier was designed, where the user has to pass each task in order to gain admittance. The first task was the opening and closing of the left hand and the second task was the opening and closing of the right hand. Both tasks were evaluated by the authentication system and only if the subject passes authentication for both tasks, is entry granted. 

To increase the reliability of the system, when calculating TN and FP, we used not only imposters selected in the testing dataset, which were selected for cross-validation, but all trials from all remaining subjects (overall approximately 10,000 trials) who were not used for training. The Receiver Operating Characteristic (ROC) analysis [34] was used to show the most complete picture of the authentication system as it depicts the relation between TPR (=1 − FRR) and FAR. An additional parameter is FAR for each registered user.

## 3. Results

Classification accuracies, FAR and FRR were calculated for systems with 8, 16, and 64 channels for PCA-SVM and two types of NN-SVM, and the results listed in Table 1. 

**Figure 6:**
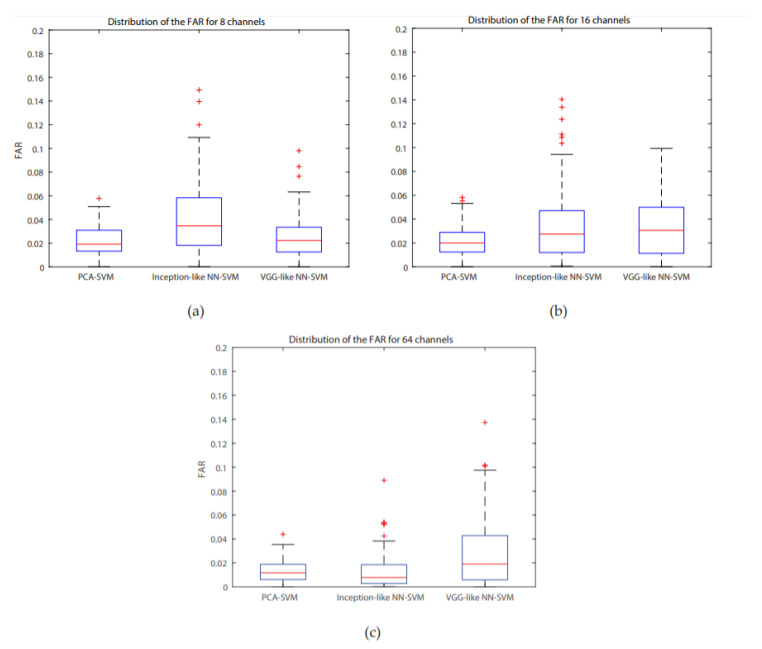
Distribution of FAR across subjects for the PCA-SVM, inception-like NN-SVM, VGG-like NN-SVM (**a**) 8 channels, (**b**) 16 channels, (**c**) 64 channels.

**Figure 7:**
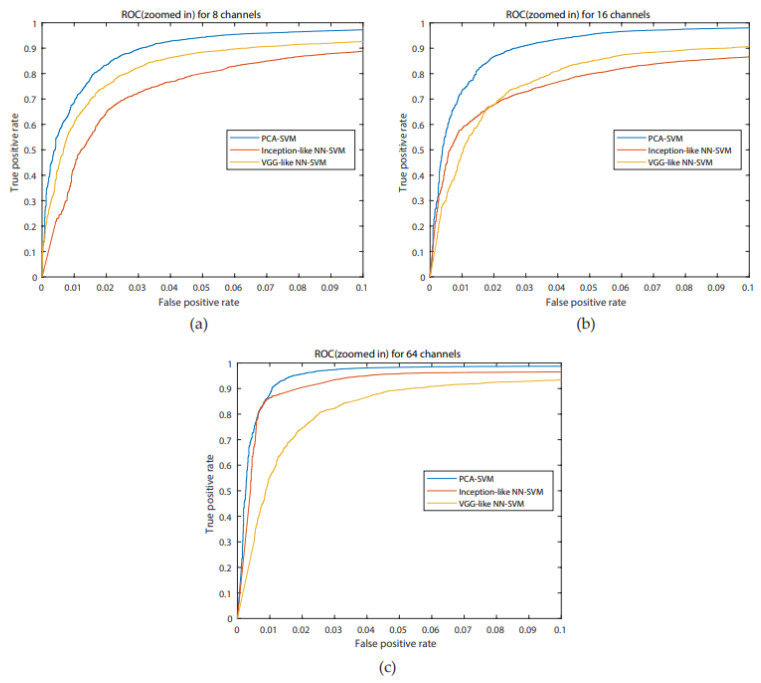
ROC curves for the PCA-SVM (blue), inception-like NN-SVM (red), VGG-like NN-SVM (yellow) for a false positive rate (FAR) ranging from 0 to 0.1 to emphasize differences for 8 channels (**a**), 16 channels (**b**), 64 channels (**c**).

**Figure 8:**
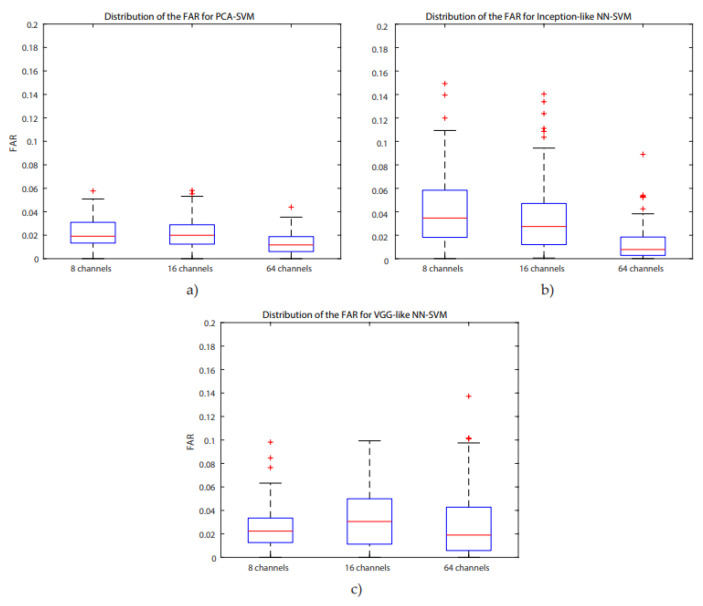
Distribution of FAR across subjects for 8, 16, and 64 channels for the (**a**) PCA-SVM, (**b**) inception-like NN-SVM, and (**c**) VGG-like NN-SVM.

**Figure 9:**
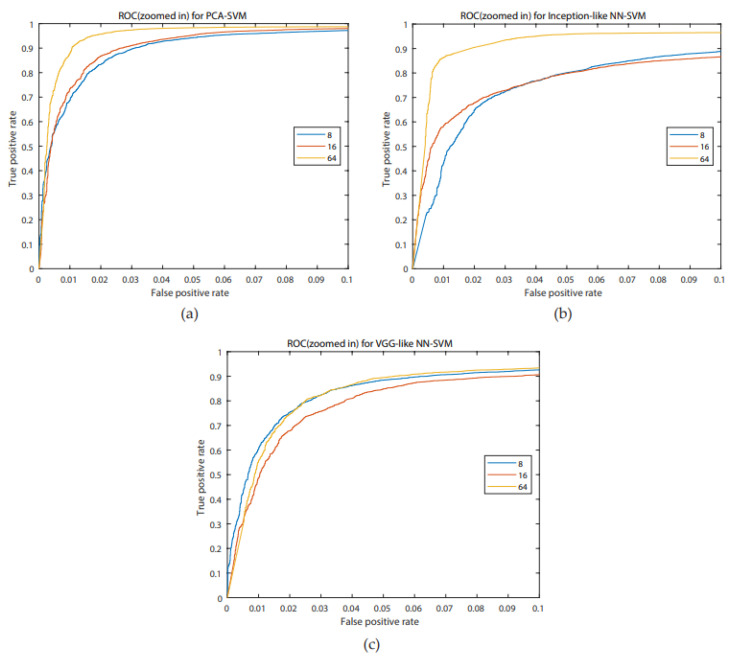
ROC curves for the PCA-SVM (blue), inception-like NN-SVM (red), VGG-like NN-SVM (yellow) for a false positive rate (FAR) ranging from 0 to 0.1 to emphasize differences ranging from 0 to 0.1 for 8 channels (**a**), 16 channels (**b**), 64 channels (**c**).

For all systems, accuracies are higher than 85%, the highest one is for the PCA-SVM system for 64 channels (95.64%), with a concurrent maximum value for the FAR. A Bonferroni corrected (for multiple comparisons, α = 0.05/3) Wilcoxon signed rank test with H0 being the compared populations sharing a distribution with an identical median was performed for the FAR results. It showed that for 8 channels, the inception-like NN-SVM performed significantly worse than VGG-like NN- SVM (*p* = 4.756 × 10^−8^) and the PCA-SVM (*p* = 3.539 × 10^−11^). The VGG-like NN-SVM and PCA-SVM performed equally well when evaluating the FAR. For 16 channels, the PCA-SVM significantly outperformed the inception-like NN-SVM (*p* = 1.215 × 10^−5^) and the VGG-like NN-SVM (*p* = 1.864 × 10^−5^) whilst the two NN-SVM systems performed equally. For 64 channels, the VGG-like NN-SVM was significantly outperformed by the PCA-SVM (*p* = 2.228 × 10^−7^) and by the inception-like NN-SVM (*p* = 1.514 × 10^−8^) whilst the PCA-SVM and inception-like NN-SVM performed equally. Boxplots of the FAR distribution of the different systems for the same number of channels can be found in Figure 6. ROC- curves of all models are presented in Figure 7. We investigated similarly the FAR of the systems when more channels are considered. For the PCA-SVM system, drawing features from 64 channels had a significant effect on FAR (*p* = 3.541 × 10^−13^) compared to 8 channels and (*p* = 7.915 × 10^−17^) to 16 channels. Similarly, the FAR of the inception-like NN-SVMs for 64 channels outperformed 8 channels (*p* = 7.915 × 10^−17^) and 16 channels (*p* = 2.689 × 10^−12^). There was no significant difference between the FARs of the VGG-like NN. Boxplots of the FAR distribution of the different systems for the same number of channels can be found in Figure 8. ROC-curves of all models are presented in Figure 9.

## 4. Discussion

When comparing the results, we can conclude that the FAR of the PCA-SVM and inception-like NN-SVM systems significantly decreases with the number of channels whilst not significantly affecting the FAR of the VGG-like NN-SVM. A possible reason is that authentication, which in our case relies on motor movement, also benefits from channels not connected with motor movement activity. Also note that, when combining a neural network with an SVM, accuracy increases with the number of channels more substantially when a deeper NN is considered. In [29] the authors concluded that a shallower neural network is more optimal for EEG analysis whereas the authors of [35] reported a mixture of successes with both deep and shallow neural networks, albeit they admitted that more research was required. As to our work, it seems that the deeper network, i.e., the inception-like NN benefits more from the increase in channels than the VGG-like NN.

Second, we conclude a similar, slightly inferior accuracy with respect to the state-of-the-art results with the PCA-SVM system. We wish to point out that the accuracies in our work are for single trials whilst a multitude of published reports relied on repeated trials. The number of subjects in the database we used was also higher than that used in the mentioned studies.

Third, there is the training time difference between the suggested models. Using a standard laptop with an intel core i7 (6th generation) without additional graphical cards, the PCA-SVM system required approximately 30 min to perform training of a new decoder for a user, the VGG-like NN- SVM system on average 1 h and the inception-like NN-SVM system on average 3 h. The added complexity of the NN systems require more computational power and time than their simpler counterparts. The removal of users does not require retraining of the systems for other users.

We are aware of the following limitations of our work:

1. For a fair comparison, the methods implemented in published reports should be tested on the dataset used in this work. To the best of our knowledge, we could not find any code online. This motivated us to make our code publicly available to encourage future comparisons.
2. The application of EEG electrodes is quite time consuming and a hurdle in the adoption of EEG- based authentication. We aim to investigate the usage of dry electrode EEG recordings.
3. The addition of a new user requires training of an authentication system for that user.
4. The system should be tested using data collected on different days and recording sessions to show robustness.

## 5. Conclusions

We propose an EEG-based decoder capable of authenticating an individual against a sizeable population of imposters. For the proposed decoder, we considered both a combination of an inception-like NN, VGG-like NN or PCA with an SVM classifier, for different subgroups of channels. Taking the FAR as the most important measure, the PCA-SVM outperforms the inception-like NN- SVM combination significantly for 8 and 16 channels with equal performance for 64 channels. The VGG-like NN-SVM performed equal to the PCA-SVM for 8 channels but performed significantly worse on 16 and 64 channels. The FAR of the PCA-SVM and inception-like NN-SVM increased significantly with the number of channels. For real-world applications, we recommend the simpler, faster, and more accurate PCA-SVM.

## Figures and Tables

**Table 1:** Accuracies, FRR and FAR for 8-channel models, 16-channel models, 64-channel models using the optimal threshold. The standard deviation (std) can be found between brackets and the area under curve (AUC) the area under the ROC curve.

	8 channels			
	Accuracy (std)	FRR (std)	FAR (std)	AUC
*PCA-SVM*	0.9209 (0.0442)	0.1355 (0.0798)	0.0219 (0.0122)	0.9809
*VGG-like* *NN-SVM*	0.8829 (0.0599)	0.2027 (0.1072)	0.0255 (0.0180)	0.9632
*Inception-like* *NN SVM*	0.8728 (0.0615)	0.2231 (0.1065)	0.0408 (0.0301)	0.9402
	16 channels			
	Accuracy (std)	FRR (std)	FAR (std)	AUC
*PCA-SVM*	0.9236 (0.0461)	0.1302 (0.0794)	0.0217 (0.0128)	0.9843
*VGG-like* *NN-SVM*	0.8747 (0.0743)	0.2162 (0.1298)	0.0333 (0.0256)	0.9533
*Inception-like* *NN SVM*	0.8531 (0.0789)	0.2605 (0.1320)	0.0353 (0.0314)	0.9396
	64 channels			
	Accuracy (std)	FRR (std)	FAR (std)	AUC
*PCA-SVM*	0.9564 (0.0285)	0.0737 (0.0481)	0.0126 (0.0090)	0.9891
*VGG-like* *NN-SVM*	0.9068 (0.0580)	0.1636 (0.1020)	0.0289 (0.0290)	0.9586
*Inception-like* *NN SVM*	0.9340 (0.0314)	0.1240 (0.0569)	0.0127 (0.0144)	0.9755

## Supplementary Materials

The following are available online at https://www.mdpi.com/2079-6374/10/9/124/s1, Figure S1: Modules A and C are intended for extending the number of feature maps, allowing the network to go deeper and increase performance. Module B is intended for the reduction of grid size. Modules A, B, and C are only used in the inception-like NN, Figure S2: Convolution in the inception-like NN, Figure S3: Structure of individual blocks of the VGG-like network, Figure S4: ROC curves for the 8 channels (blue), 16 channels (red), 64 channels (yellow) for FAR ranging from 0 to 1 for PCA-SVM (a), inception-like NN-SVM (**b**), VGG-like NN-SVM (c), Figure S5: Mean cross-correlation of all features and standard deviation of the mean for 8 channels (a,b) and 16 channels (c,d), Figure S6: Representation of 8 (a) and 16 (b) selected channels.

## Code

The code of the project is available online at https://github.com/UladzislauBarayeu/biosensors-10-00124.
