# Supplementary material for: Robust Single-Trial EEG-Based Authentication Achieved with a 2-Stage Classifier
Source: Biosensors (Basel). 2020 Sep 13;10(9):124. doi: 10.3390/bios10090124 (PMC7558120; doi:10.3390/bios10090124)
Supplement: Supplementary file 1 [file biosensors-10-00124-s001.pdf]

biosensors

MDPI

Article

Robust Single-Trial EEG-Based Authentication Achieved with a 2-Stage Classifier

Uladzislau Barayeu [1,†], Nastassya Horlava [2,†], Arno Libert [3,*] and Marc Van Hulle [3,*]

1 Department of Biophysics, Belarusian State University, Nesavisimosti Ave., 4, 220030 Minsk, Republic of Belarus; baraewlad@gmail.com;
2 Department of Mathematical Modelling and Data Analysis, Belarusian State University, Nesavisimosti Ave., 4, 220030 Minsk, Republic of Belarus; nastassya.horlava@mailbox.tu-dresden.de
3 Laboratory for Neuro- and Psychophysiology, Department of Neuroscience, KU Leuven, O&N2, Herestraat 49, 3000 Leuven, Belgium; arno.libert@kuleuven.be
* Correspondence: marc.vanhulle@kuleuven.be
† These authors contributed equally to this work.

Received: 30 June 2020; Accepted: 11 September 2020; Published: date

Supplementary Material

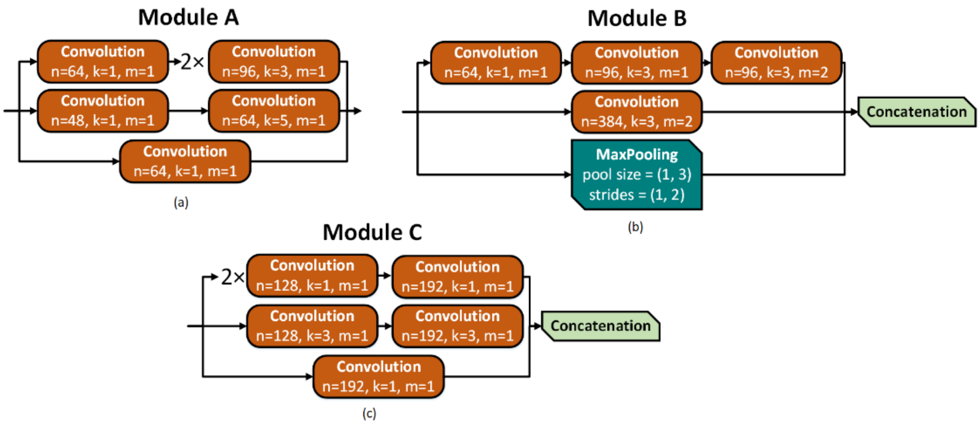

Figure S1: Modules A and C are intended for extending the number of feature maps, allowing the network to go deeper and increase performance. Module B is intended for the reduction of grid size. Modules A, B and C are only used in the inception-like NN.

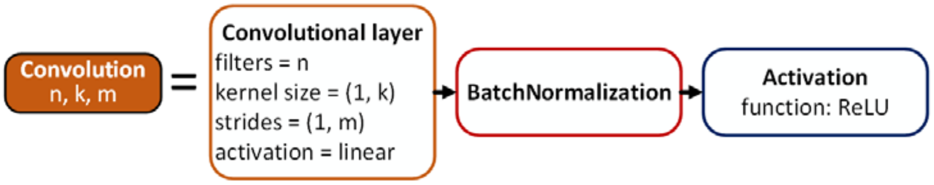

Figure S2: Convolution in the inception-like NN.

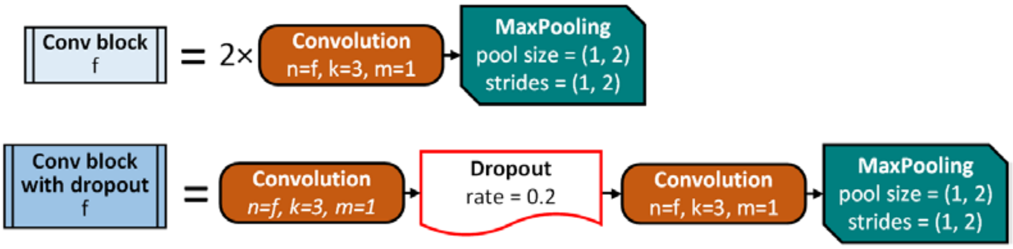

Figure S3: Structure of individual blocks of the VGG-like network.

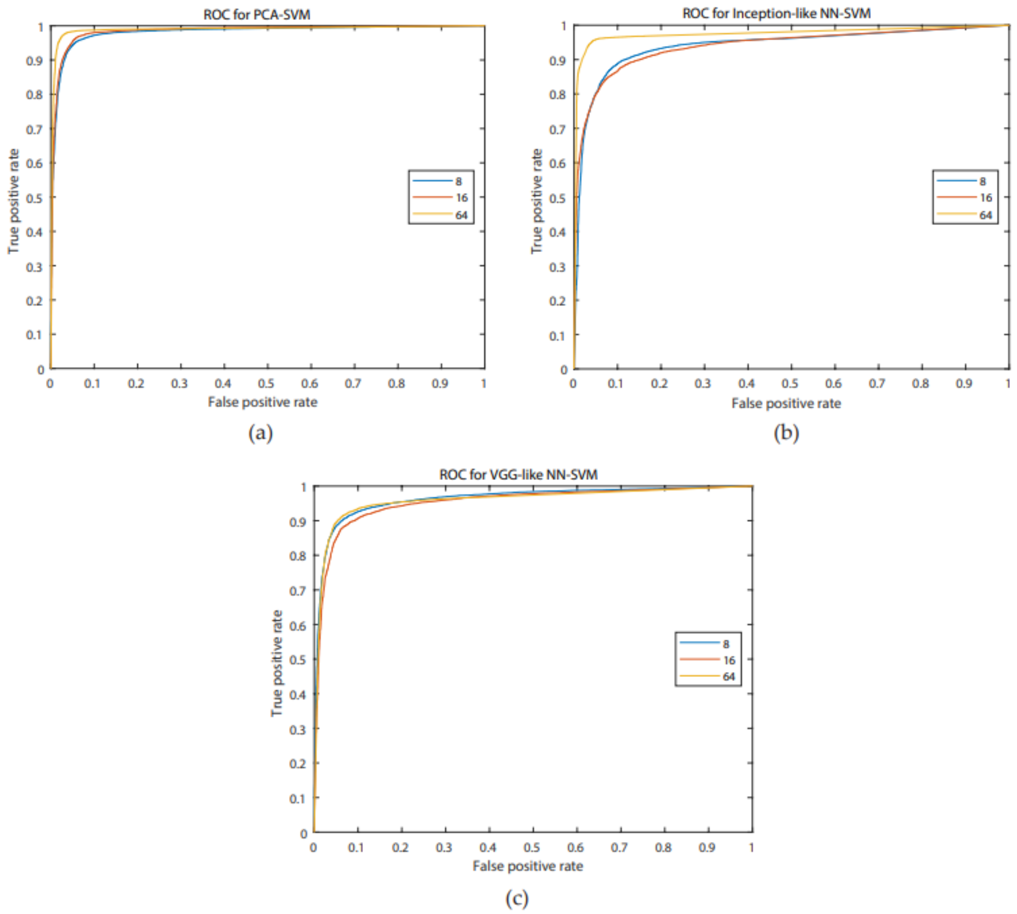

Figure S4. ROC curves for the 8 channels (**blue**), 16 channels (**red**), 64 channels (**yellow**) for FAR ranging from 0 to 1 for PCA-SVM (**a**), inception-like NN-SVM (**b**) VGG-like NN-SVM (**c**).

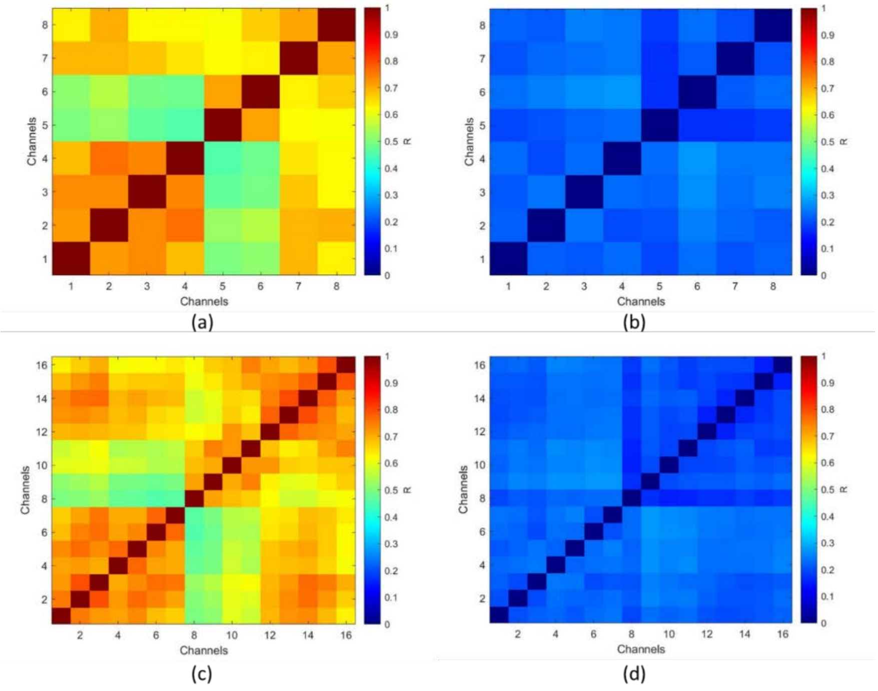

Figure S5: Mean cross-correlation of all features and standard deviation of the mean for 8 channels (**a**, **b**) and 16 channels (**c**, **d**).

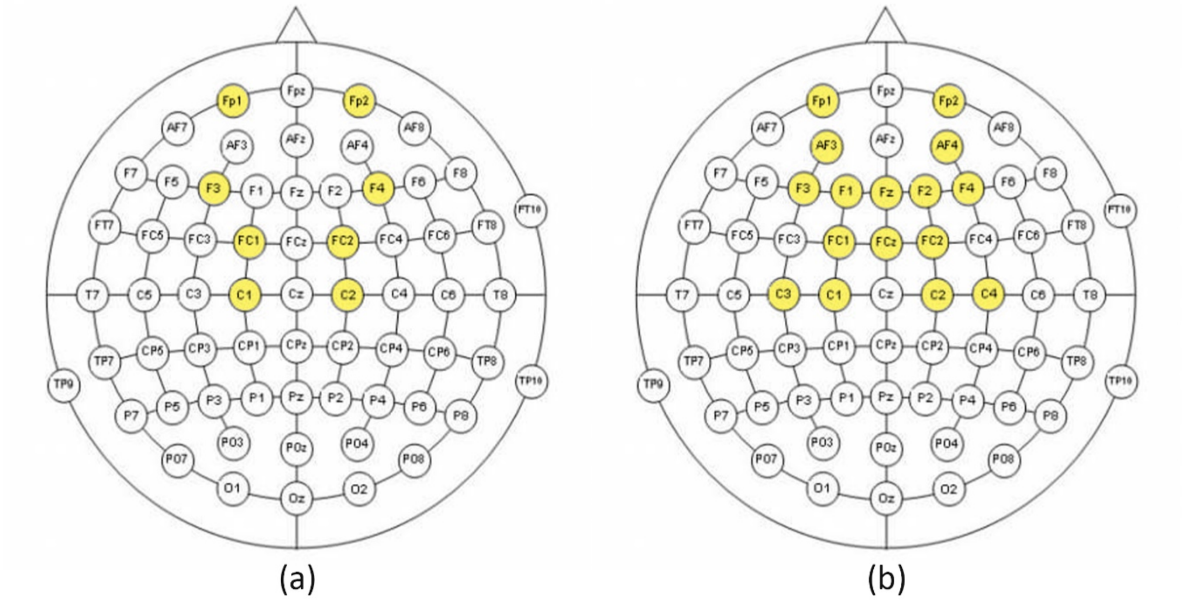

Figure S6: Representation of 8 (**a**) and 16(**b**) selected channels.

© 2020 by the authors. Licensee MDPI, Basel, Switzerland. This article is an open access article distributed under the terms and conditions of the Creative Commons Attribution (CC BY) license (http://creativecommons.org/licenses/by/4.0/).
